# All-cause mortality supports the COVID-19 mortality in Belgium and comparison with major fatal events of the last century

**DOI:** 10.1186/s13690-020-00496-x

**Published:** 2020-11-13

**Authors:** Natalia Bustos Sierra, Nathalie Bossuyt, Toon Braeye, Mathias Leroy, Isabelle Moyersoen, Ilse Peeters, Aline Scohy, Johan Van der Heyden, Herman Van Oyen, Françoise Renard

**Affiliations:** 1 Scientific Directorate of Epidemiology and public health, Sciensano, J.Wytsmanstraat 14, 1050 Brussels, Belgium; 2 Public Health and Primary Care, Faculty of Medicine, Ghent University, Ghent, Belgium

**Keywords:** COVID-19, Mortality, All-cause mortality, Excess mortality, Pandemic

## Abstract

**Background:**

The COVID-19 mortality rate in Belgium has been ranked among the highest in the world. To assess the appropriateness of the country’s COVID-19 mortality surveillance, that includes long-term care facilities deaths and deaths in possible cases, the number of COVID-19 deaths was compared with the number of deaths from all-cause mortality. Mortality during the COVID-19 pandemic was also compared with historical mortality rates from the last century including those of the Spanish influenza pandemic.

**Methods:**

Excess mortality predictions and COVID-19 mortality data were analysed for the period March 10th to June 21st 2020. The number of COVID-19 deaths and the COVID-19 mortality rate per million were calculated for hospitals, nursing homes and other places of death, according to diagnostic status (confirmed/possible infection). To evaluate historical mortality, monthly mortality rates were calculated from January 1900 to June 2020.

**Results:**

Nine thousand five hundred ninety-one COVID-19 deaths and 39,076 deaths from all-causes were recorded, with a correlation of 94% (Spearman’s rho, *p* < 0,01). During the period with statistically significant excess mortality (March 20th to April 28th; total excess mortality 64.7%), 7917 excess deaths were observed among the 20,159 deaths from all-causes. In the same period, 7576 COVID-19 deaths were notified, indicating that 96% of the excess mortality were likely attributable to COVID-19. The inclusion of deaths in nursing homes doubled the COVID-19 mortality rate, while adding deaths in possible cases increased it by 27%. Deaths in laboratory-confirmed cases accounted for 69% of total COVID-19-related deaths and 43% of in-hospital deaths. Although the number of deaths was historically high, the monthly mortality rate was lower in April 2020 compared to the major fatal events of the last century.

**Conclusions:**

Trends in all-cause mortality during the first wave of the epidemic was a key indicator to validate the Belgium’s high COVID-19 mortality figures. A COVID-19 mortality surveillance limited to deaths from hospitalised and selected laboratory-confirmed cases would have underestimated the magnitude of the epidemic. Excess mortality, daily and monthly number of deaths in Belgium were historically high classifying undeniably the first wave of the COVID-19 epidemic as a fatal event.

**Supplementary Information:**

The online version contains supplementary material available at 10.1186/s13690-020-00496-x.

## Background

On January 30th 2020, after the spread of the coronavirus outbreak beyond the Chinese borders and the notification of the first cases of coronavirus disease 2019 (COVID-19) in Europe, the COVID-19 epidemic was declared a Public Health Emergency of International Concern by the WHO [1]. In the days that followed, a group of nine Belgian nationals residing in Wuhan, China, were repatriated to Belgium and quarantined in a military hospital. Amongst them, one asymptomatic person tested positive for the severe acute respiratory syndrome coronavirus 2 (SARS-CoV-2) on February 3rd. A month later, at the end of the spring mid-term holidays, fear of new cases arising in returning holidaymakers proved to be well-founded. Indeed, the first wave of the epidemic began on March 1st with a second confirmed infection in a traveller returning from the Oise region, France. The next day, four additional cases, returning from Italy, were confirmed. By March 10th, Belgium had recorded 601 cases and reported the first COVID-19-related death in a 90-year-old woman [2]. Schools and universities closed on March 16th and the official lockdown began on March 18th. By June 21st, end date of the country’s first wave, 9591 deaths had been recorded and the mortality rate of COVID-19 in Belgium was among the highest worldwide [3]. However, this high ranking could partly result from the case definition of COVID-19-related deaths used in the country. Indeed, in addition to laboratory-confirmed cases, possible cases and radiologically confirmed cases were included in the official Belgian figures, which was not the case in other European countries [4]. Moreover, Belgium not only registered deaths that occurred in hospitals, but also those in the wider community, including long-term care facilities (LTCF) and at home [5].

To assess the appropriateness of Belgium’s COVID-19 mortality surveillance, this article compares the number of COVID-19 deaths with the number of deaths from all causes during the first wave of the epidemic. To better apprehend the weight of place of death and diagnostic status (laboratory-confirmed, radiologically-confirmed, possible case) on the overall death numbers, the COVID-19 mortality rate per million is broken down according to these variables. Finally, mortality during the first wave of the COVID-19 pandemic is compared with historical mortality rates from the last century, including those of the Spanish influenza pandemic.

## Methods

### COVID-19 mortality

Daily COVID-19 numbers of deaths are reported by Sciensano, the Scientific Institute of Public Health in Belgium using reports from hospitals, LTCFs (including mainly nursing homes (NH), residence services for elderly persons, facilities for disabled persons), and general practitioners. For each place of death, the diagnostic status (confirmed by RT-PCR or chest CT scan [6] and possible cases) is reported. Possible cases are those who meet the clinical criteria for the disease [7] but do not undergo a diagnostic test, or whose diagnostic tests are inconclusive or negative, whether or not there is an epidemiological link to a confirmed case. For both possible and confirmed cases, if a clear alternative cause of death that cannot be linked to COVID-19 (e.g. trauma) is identified, the death is not included in the surveillance. The mortality surveillance methodology follows the ECDC and WHO guidelines [8, 9]. However, the definition of a probable case is not used because this definition, at first, concerned people with an inconclusive test, which was infrequently observed in Belgium. Radiologically-confirmed cases were added because of the imperfect sensitivity of the RT-PCR tests, and periods of limited access to testing due to shortage of reagent or swab or delay in obtaining results. Most COVID-19 deaths are notified within two calendar days and published according to the date of death. Details of the COVID-19 mortality surveillance in Belgium is described by Renard et al. [10]. The chronology of the COVID-19 case definition and testing strategy is provided in Supplementary Table 1.

### All-cause mortality

The all-cause mortality by day is provided weekly by the National Register to the Infectious Diseases Epidemiology Unit of Sciensano in the framework of the Be-MOMO project (the Belgian Mortality Monitoring) [11]. Data are available from January 1989 onwards. Be-MOMO is designed to serve as a tool for early detection and quantification of unusual mortality that could result from disease outbreaks or extreme environmental conditions. Around 95% of mortality data are available after two weeks. Observed death counts are compared to expected deaths and a threshold defining excess mortality, both obtained by modelling the past five years of mortality data. Expected deaths are the model predictions and represent normal/average mortality levels. They are used for the calculation of the excess number of deaths (observed – expected). The threshold defining an excess mortality is the upper limit of the prediction interval around expected mortality, calculated by a 2/3-power transformation to correct for skewness in the Poisson distribution [12]. Threshold values represent critical mortality levels and are used to detect unusual or significant excess mortality. The confidence level for the upper threshold is chosen as the optimal compromise between sensitivity and specificity of alert detection. It is set at 99.5% for daily mortality data. To model the complete five years’ time series and reduce random variation in the predicted baseline for daily-level data, a sine and cosine wave component is added to capture the seasonal pattern of mortality. The methodology of Be-MOMO is described by Cox et al. [13].

### Historical reconstitution of the all-cause mortality

To assess the historical mortality, monthly mortality rates per 100,000 inhabitants are calculated from January 1900 to June 2020, using the population as of January 1st of each year. A monthly average is calculated for years where only annual figures are available (from 1900 to 1949). From January 1950 to 1995 and for six specifics calendar years (1920, 1925, 1930, 1935, 1940, and 1945), the number of deaths is available by month. The unavailability of age at death for people who died before 1989 did not allow us to standardize mortality rates by age. Statistics Belgium provided the number of deaths before 1989 and the population sizes. Be-MOMO, based on data from the National Register provided the number of deaths from January 1989.

### Mortality in 2020

The comparison between COVID-19 mortality and all-cause mortality is made for the first COVID-19 epidemic wave (March 10th to June 21st). A Spearman test [14] is used to test the daily correlation between these two variables. Daily all-cause mortality was extracted on August 22th 2020. Daily COVID-19 mortality was extracted on August 27th. COVID-19 mortality rate per million is calculated according to places of death (hospitals, NHs and other settings) in relation to the different diagnostic status (laboratory-confirmed cases by RT-PCR, chest CT scan, and possible cases), using the Belgian population as of January 1st 2020 (*N* = 11,492,641 inhabitants). The comparison between COVID-19 and all-cause mortality by age groups is available in the supplementary material [see Additional file 2].

### Statistical software

The data analysis and figures were performed using SAS Software and R Studio.

## Results

### COVID-19 deaths, all-cause mortality, and excess mortality during the 1st wave of COVID-19 in 2020

Between March 10th to June 21st 2020, there were 9591 COVID-19 deaths and 39,076 deaths from all-causes. Four weeks after the start of the lockdown, on April 8th, the peak of COVID-19 mortality was reached with a daily total of 321 deaths. Two days later, a peak of all-cause mortality was observed (April 10th: 669 deaths). Correlation between the daily numbers of all-cause mortality and COVID-19 mortality was 94% (Spearman’s rho, *p* < 0,01). A period of statistically significant excess mortality was observed from March 20th to April 28th. There was no significant excess mortality between April 29th and May 4th. Subsequently, three additional days of significant excess mortality were observed: May 5th, 8th^,^ and 9th. Of the 20,159 all-cause deaths observed between March 20th and April 28th, Be-MOMO estimated an excess of 7917 deaths, representing 64.7% excess mortality (12,242 expected deaths). In this period, the number of excess deaths from all causes was almost equal to the number of COVID-19 deaths (7576), indicating that 96% of the excess mortality were likely attributable to COVID-19 or to the health crisis it created. Excess mortality was highest among people over 85 years of age (78.9%), with 96% of this excess mortality also attributable to COVID-19. An additional table shows mortality for the age groups 0–64, 65–84 and 85+ [Supplementary Table 2].

### Case definition and COVID-19 mortality rate

By June 21st, 49% of the COVID-19 deaths had occurred in hospitals, 50% in NHs, and 1% at home or in other residential communities. Two-thirds of COVID-19 deaths involved NH residents counting those who died in hospitals. In total, 69% of the COVID-19 deaths were laboratory-confirmed, 4% were confirmed by chest CT scan, and 27% were defined as possible cases (Table 1). In hospitals, the most frequent diagnostic status was laboratory confirmed (87%), followed by chest CT scan confirmed and possible cases, accounting respectively for 8 and 5% of deaths. During the first weeks of the epidemic, laboratory tests were not routinely available in NHs, explaining a high percentage of deaths classified as possible cases in these facilities (48%).

**Table 1:** COVID-19 deaths and mortality rate per million inhabitants according to place of death and diagnostic status, March 10th to June 21st 2020, Belgium

Place of death	Diagnostic status	Number of COVID-19 deaths	COVID-19 mortality rate per million	% COVID-19 number of deaths by place of death	% COVID-19 number of deaths by all places of death
Hospital	Laboratory confirmed cases	4114	358.0	87%	43%
	Chest CT scan cases	385	33.5	8%	4%
	Possible cases	230	20.0	5%	2%
	**Total cases**	**4729**	**411.5**	**100%**	**49%**
Nursing home	Laboratory confirmed cases	2450	213.2	52%	26%
	Chest CT scan cases	4	0.3	0%	0%
	Possible cases	2299	200.0	48%	24%
	**Total cases**	**4753**	**413.6**	**100%**	**50%**
Other place	Laboratory confirmed cases	53	4.6	49%	< 1%
	Chest CT scan cases	1	0.1	1%	0%
	Possible cases	55	4.8	50%	< 1%
	**Total cases**	**109**	**9.5**	**100%**	**1%**
All places	Laboratory confirmed cases	6617	575.8	69%	
	Chest CT scan cases	390	33.9	4%	
	Possible cases	2584	224.8	27%	
	**Total cases**	**9591**	**834.5**	**100%**	**100%**

**Fig. 1:**
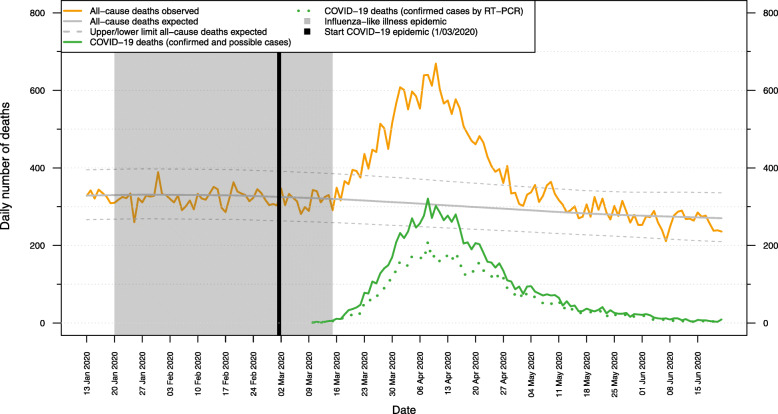
Mortality all-cause (Be-MOMO) and related to COVID-19, March 10th to June 21st 2020, Belgium. How to read this graph? When the number of deaths per day (orange line) exceeds the upper or lower limits of the deaths predicted by the modelling (grey dashed lines), there is a significant excess or under-mortality. The green curve corresponds to the daily number of COVID-19 deaths (all diagnostic status and all places of death). The green dotted line represents laboratory-confirmed COVID-19 deaths (all places of death)

The COVID-19 mortality rate was 834.5 deaths per million. The inclusion of deaths of confirmed and possible cases that occurred in NHs (respectively 26 and 24% of the total COVID-19 death figures) doubled the COVID-19 mortality rate (+ 413.6 deaths per million). The inclusion of chest CT scan positive cases had only a slight effect (+ 33.9 deaths per million, 4% of the total figures). If considering only laboratory-confirmed deaths occurring in hospitals, mortality rate would have been calculated at 358 deaths per million (43% of the total COVID-19 deaths in Belgium). When including both the in and out-of-hospital deaths, mortality rate in laboratory-confirmed cases was 575.8 per million (69% of total COVID-19 deaths, green dotted line, Fig. 1). The inclusion of deaths in possible cases into the national mortality surveillance helped raise awareness of the particularly difficult situation occurring in NHs. A mass screening campaign of all residents and staff in NHs implemented early April resulted in an increase in the proportion of deaths from confirmed cases at the expense of possible cases, as shown in Fig. 1.

Figure 1 is an overview of Belgium’s mortality results. Key results are: 1) a substantial statistically significant excess mortality, 2) a high correlation between COVID-19 mortality and excess all-cause mortality 3) surveillance of laboratory-confirmed COVID-19 deaths alone would have underestimated the number of COVID-19-related deaths by approximately 30%. An additional figure shows mortality for the age groups 0–64, 65–84 and 85+ [see Additional file 2].

### The history of excess mortality

#### Number of deaths

For the period 2015–19, Belgium had a seasonal mortality pattern with an average of 321 deaths per day during winter (weeks 41 to 19) against 270 during summer (weeks 20 to 40). The highest number of daily deaths in this period was recorded on March 7th 2018, at 465 deaths, coinciding with the peak of the ongoing flu epidemic. Current excess mortality increased from March 20th 2020 onwards with a high number of daily deaths for a prolonged period. During 21 days (between March 27th and April 17th), over 500 deaths per day were observed, including seven days with over 600 deaths. Belgium observed 15,398 deaths from all causes for April 2020 compared to an average of 8854 deaths for the same month in 2015–2019. The monthly mortality rate per 100,000 for April 2015 and 2019 were respectively 81.4 and 77.3, compared to 134 in April 2020.

Number of deaths per day are available since 1989. Between 1989 and 2020, the only other event in which Belgium experienced more than 500 deaths per day was during the 1989 influenza A(H3N2) epidemic, with two days in mid-December with 516 and 520 deaths [15].

The number of monthly deaths during the COVID-19 pandemic was higher than the number of monthly deaths observed during the 1968–1970 influenza pandemic. At that time, the Hong Kong influenza A(H3N2) virus returned to Europe by the end of 1969 after an initial decrease in the number of cases [16]. During this second wave, Belgium recorded 13,333 and 14,255 deaths in December 1969 and January 1970 respectively.

The number of deaths in April 2020 was also similar to that of the flu epidemics of January 1951 and February 1960, which claimed about 15,500 deaths per month [17, 18]. These numbers have only been surpassed by the number of deaths recorded at the beginning and towards the end of the Second World War (May 1940, 23,106 deaths; January 1945, 15,950 deaths) and most probably by the Spanish influenza A(H1N1) pandemic, although monthly figures of this specific period are not available.

The Spanish influenza claimed the lives of nearly 50 million people worldwide. Between April 1918 and the spring of 1919, the pandemic rolled out three deadly waves [19]. At the end of 1918, the second wave caused more deaths worldwide than the First World War, and the third wave, which heavily affected the southern hemisphere, had more casualties than the first wave. In Belgium, 157,340 deaths were recorded in 1918, higher than the average of 108,815 deaths per year recorded during the First World War, and suggesting that approximately 50,000 additional deaths were likely attributable to influenza [20].

#### Mortality rates

**Fig. 2:**
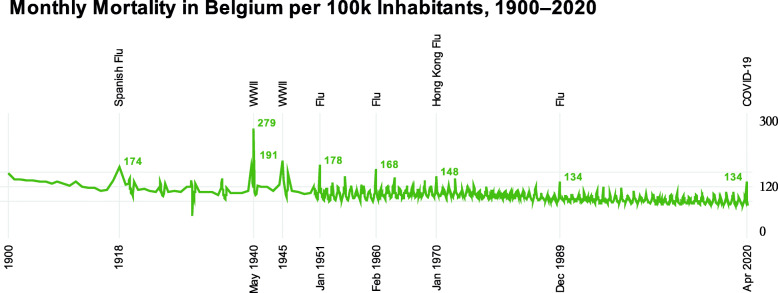
The monthly mortality rate per 100,000 inhabitants from 1900 to 2020, Belgium

Figure 2 shows peaks in the monthly mortality rate related to the aforementioned events from the past century. The monthly mortality rate in April 2020 in Belgium (134 per 100,000) was equal to the rate of the 1989 influenza epidemic, close to the 1968–1970 Hong Kong influenza rate (147 per 100,000 in January 1970), but lower than those observed at the start and the end of the Second World War (respectively 279 and 191 per 100,000) and during the Spanish influenza pandemic. The average monthly mortality rate in 1918 was 174 per 100,000 inhabitants. In Belgium, the Spanish influenza excess mortality mainly occurred in the last quarter of 1918 [21]. Thus, the average monthly mortality rate reported for that year is likely to underestimate the mortality rates from October to December.

## Discussion

### COVID-19 deaths, all-cause mortality, and excess mortality during the 1st wave of COVID-19 in 2020

The excess mortality in the Belgian population recorded between March and May 2020 is clearly related to the health crisis caused by COVID-19, as shown by the proportion of people who died in this period as a direct result of SARS-CoV-2 infection. The inclusion of deaths in possible cases and deaths occurring outside hospital settings in our surveillance resulted in a recorded number of COVID-19 deaths very close to the number of excess deaths calculated by Be-MOMO. In Belgium, similar to the 2018–19 flu season, no excess mortality was observed during the 2019–20 flu epidemic (January 20th - March 15th 2020), suggesting that many vulnerable people were still alive when the COVID-19 epidemic hit. Two-thirds of the deaths occurred in NH residents indicating that COVID-19 deaths were strongly related to the number of NHs affected by the virus [22]. During the first wave, 89% of NHs in Belgium reported at least one possible or confirmed COVID-19 case, and outbreaks with at least 2 cases and 10 cases were reported by 76 and 40% respectively.

Excess mortality during the COVID-19 first wave epidemic could also be linked to a delay in the management of both acute and chronic pathologies, with difficulties in access to care during lockdown and with patients hesitating to seek medical care for fear of catching the virus. Increased frailty [23] and downward spiral syndrome (or geriatric failure to thrive) in the elderly, as a result of social isolation and reduced physical activity, may have also played a key role [24]. Also, the impact of the crisis on psychological well-being may have increased death from self-harm, and lockdown those of home accidents [25]. On the other hand, confinement certainly reduced mortality linked to traffic, sports, nightlife, or work accidents for younger age groups, and limited population exposure to pollutants, a known driver of excess mortality. Not all deaths related to the COVID-19 can be considered excess mortality, as a small portion of these deaths would likely have occurred at that time as part of the expected number of deaths. But no short-term harvest effects (a statistically significant lower number than expected daily deaths) were observed after the peak, suggesting that the majority of those who died would not have died in the short term. An analysis of the causes of death will be required to validate these hypotheses, but these data, taken from death certificates, are generally available in Belgium with a delay of 24 months.

We assert that the all-cause mortality data are consistent with the high number of deaths related to COVID-19. Indeed, the difference between the total number of deaths and the COVID-19 deaths represented about 300 deaths per day, the usual daily average in Belgium in April. Moreover, the first wave of the COVID-19 epidemic took place at a time when there is usually no excess mortality in the country. Excess mortality in Belgium is generally limited to periods of seasonal influenza, cold waves, smog (December to mid-March), heat-waves and ozone peaks (June to August). In April, no other factor than the appearance of COVID-19 was identified as a likely cause to explain the level of excess mortality recorded [26]. On the 8th and 9th of May, high ozone concentrations were recorded with 137 and 131 μg/m^3^ respectively (https://www.irceline.be/en), with a threshold set at 100 μg/m^3^ by WHO for the daily highest 8-h mean concentration. Be-MOMO does not allow to attribute a cause to the excess mortality, but these ozone peaks may have contributed to the excess mortality in the short term, as is common to observe during the summer [27].

### Case definition and COVID-19 mortality rate

Case identification and testing policies were and remain affected by the reality of logistical constraints, especially at the onset of the crisis. Similar to other countries, the Belgian authorities regularly adapted the COVID-19 case definition and criteria for RT-PCR testing (Supplementary Table 1). The criteria evolved according to the COVID-19 epidemiology but also to the availability of personal protective equipment, swab sticks, laboratory kits, and capacity [28]. The initial testing strategy was limited to people who had an epidemiological link with areas with intense local transmission within the last 14 days and who presented severe respiratory symptoms. From March 11th onwards, tests were reserved for hospitalised people and healthcare workers (HCW) with respiratory symptoms. As the number of people to be tested exceeded capacity, the procedure for testing HCWs was restricted to those who had a fever in addition to the respiratory symptoms. With the subsequent improvement capacity, the SARS-CoV-2 testing criteria was later expanded to all persons filling the country’s case definition of a possible case (May 8th).

Following the ECDC and WHO recommendations on COVID-19 mortality surveillance [8, 9], Belgium has reported deaths from hospitalised cases, cases living in LTCFs, mainly NHs, and at home. The choice to account for deaths of possible cases was linked to the lack of testing at the beginning of the epidemic while trying to have a comprehensive view of the severity of the epidemic. An alternative approach, based on laboratory test with a sensitivity of about 70% [29], together with the initial conservative testing policy, would have clearly underestimated the magnitude of the epidemic.

The inclusion of both deaths outside hospitals and possible cases is one of the reasons why Belgium ranked among the countries with the highest specific mortality rate for COVID-19. However, the ranking is misleading due to different methods used to collect mortality data for COVID-19 in each country [4, 30]. Belgium’s COVID-19 mortality rate would be reduced by 57% if reporting had been limited to deaths in laboratory-confirmed hospitalised cases. Moreover, the COVID-19 mortality surveillance that was used in the country contributed to the identification of the burden of COVID-19 infection in NHs. At the same time, the underestimation of the number of COVID-19 cases in the general population related to the initially conservative testing strategy, has created doubt in the media and policy-makers about the accuracy of the extremely high figures of COVID-19 deaths in Belgium, especially given the negative impact on the country’s image with economic and diplomatic consequences.

With an alternative and more restricted definition, Belgium would have attracted less negative attention from the international community, but the alarming situation in NHs would likely not have been picked up. These observations have contributed to the support of NHs and the initiation of large-scale screening and testing of both resident and health-care providers of such institutions.

### The history of excess mortality

The substantial excess mortality in Belgium confirms the severe impact of the first wave of the COVID-19 epidemic. The number of deaths has not been so high since the Second World War and a few rare severe influenza episodes. However, accounting for the increasing size of the Belgian population, the COVID-19 mortality rate was not as high.

## Conclusions

Trends in all-cause mortality during the first wave of the COVID-19 epidemic was a key indicator to validate Belgium’s COVID-19 mortality surveillance methodology and the high figures that it recorded. Indeed, excess mortality numbers correlated highly with the COVID-19 recorded deaths. As such, we believe that the implementation of a specific COVID-19 mortality monitoring system in the country, including possible cases and deaths in long-term care facilities, has proven to be appropriate to allow a proper assessment of the impact of COVID-19. However, its specificities have led to misleading international comparisons, and differences in country methods for mortality data collection should be better communicated. Although the monthly number of deaths in April 2020 is exceptionally high, ranking the COVID-19 epidemic as a fatal event, the monthly mortality rate was lower than during other major fatal events of the last century.

## Supplementary Information

**Additional file 1: Supplementary Table 1.** Chronology of the COVID-19 case definition and testing strategy, January to June 2020, Belgium.

**Additional file 2: Supplementary Table 2.** COVID-19 and all-cause mortality for 0-64, 65-84 and 85+ age groups, March 20th to April 28st 2020, Belgium. **Supplementary Fig. 1.** Mortality all-cause (Be-MOMO) and related to COVID-19 for 0–64, 65–84 and 85+ age groups, Belgium.

## Data Availability

The COVID-19 mortality data collected by Sciensano is available in the mortality repository, https://epistat.wiv-isp.be/covid/. The places of death and diagnostics status of COVID-19 mortality used during the current study are available from Sciensano, https://epistat.wiv-isp.be/datarequest/index.aspx on reasonable request. All-cause mortality data is available on Statbel, https://statbel.fgov.be/en/open-data/number-deaths-day-sex-district-age

## Abbreviations

Be-MOMO: Belgian Mortality Monitoring
WHO: World Health Organization
LTCF: Long-term care facilities
NH: Nursing home
RT-PCR: Real-time polymerase chain reaction
HCW: Healthcare worker
ECDC: European Centre for Disease Prevention and Control
