# Supplementary material for: All-cause mortality supports the COVID-19 mortality in Belgium and comparison with major fatal events of the last century
Source: Arch Public Health. 2020 Nov 13;78:117. doi: 10.1186/s13690-020-00496-x (PMC7662738; doi:10.1186/s13690-020-00496-x)
Supplement: Supplementary file 1 — Additional file 1: Supplementary Table 1. Chronology of the COVID-19 case definition and testing strategy, January to June 2020, Belgium. [file 13690_2020_496_MOESM1_ESM.docx]

All-cause mortality supports the COVID-19 mortality in Belgium and comparison with major fatal events of the last century.

Additional file 1

Supplementary Table 1. Chronology of the COVID-19 case definition and testing strategy, January to June 2020, Belgium

| **Date** | **COVID-19 case definition** | **Test strategy** |
| --- | --- | --- |
| 18/01 | Travellers returning from Wuhan who develop respiratory symptoms during or 14 days after travelling. | RT-PCR test is performed according to the case definition. |
| 13/02 | All persons with respiratory symptoms during travelling in China or 14 days after returning from China; OR  All persons with respiratory symptoms starting in the 14 days after having had contact with a lab-confirmed case. |  |
| 18/02 | Every person with upper/lower respiratory symptoms (acute start of minimal one of the following symptoms: fever, cough, sore throat, myalgia, respiratory difficulties, headache, …); AND History of travel to China 14 days before illness; OR Physical contact with a lab-confirmed case in 14 days before illness. |  |
| 26/02 | - Every person with upper/lower respiratory symptoms (acute start of minimal one of the following symptoms: fever, cough, respiratory difficulties); AND History of travel to region of high transmission in 14 days before illness: China, South-Korea, Iran, 11 municipalities in Italy (*Codogno, Casapusterlengo, Castiglione d’Adda, Maleo, Fombio, Bertonico, Castelgerundo, Somaglia, San Fiorano, Terranova dei Passerini, Vo Euganeo)* OR Physical contact with a lab-confirmed case in 14 days before illness. - Every person with severe acute respiratory symptoms and/or clinical or radiological prove of pneumonia, who needs hospitalization; AND History of travel to region of high transmission in 14 days before illness: Other municipalities in Lombardia/Veneto/Emilia Romagna in Italy, Singapore and Japan. |  |
| 04/03 | - Every person with fever and clinical respiratory symptoms (cough or respiratory difficulties); AND History of travel to the region of high transmission in 14 days before illness: China, South Korea, Iran, and Italian provinces of Lombardy, Veneto and Emilia-Romagna;   OR  Physical contact with a lab-confirmed case in 14 days before illness.   - Every person with severe acute respiratory symptoms and/or clinical or radiological prove of pneumonia, who needs hospitalisation, if there is no evidence of bacterial infection. |  |
| 06/03 | *Added to previous case definition:*  Municipalities in Italy : *Piemonte, Liguria, Trentino South-Tirol, Friuli-Venezia Giulia, Valle d’Aosta and the region Marche.*  *Removed from previous case definition:*  Physical contact with a lab-confirmed case in 14 days before illness. |  |
| 11/03 | **Possible case**:  Every person with acute upper/lower respiratory symptoms  - that appear new; OR - which deteriorate if the patient shows chronic respiratory symptoms. | *Start of the testing strategy:*  1. Any person whose clinical condition requires hospitalization AND where the clinician has a suspicion of COVID-19. 2. Any member of the healthcare personnel who meets the definition of a **possible case**. |
| 13/03 |  | *Added to point 2:* AND has a fever. |
| 04/04 | *Added to previous case definition:*  **Radiologically confirmed case**: A radiologically confirmed case is a person in whom the laboratory test for COVID-19 is negative, but in whom the diagnosis of COVID-19 is made based on a suggestive clinical presentation AND a compatible CT thorax. **Confirmed case**: A person with a lab-confirmed COVID-19 infection. | *Added to point 2:* Who come into contact with people at risk of a serious form of COVID-19. 3. The first cases (max 5) in a residential collectivity (nursing home, prison,…) who meet the definition of a possible case. |
| 16/04 |  | *Removed from point 2:* Fever. |
| 22/04 |  | 1. Any person for whom hospitalisation is required, including an initial admission to the day hospital.  2. Any new resident of a residential entity (e.g. residential care centre, residential centre for the disabled, prison, …). 3. Each possible case in a residential entity (if ≥2 contact region). 4. Each possible case with a member of the health care staff (persons providing care or assistance). |
| 08/05 | **Possible case:** A person with  - at least one of the following main symptoms: cough, dyspnea, thoracic pain, acute anosmia or dysgeusia without obvious cause; OR  - at least two of the following symptoms: fever, muscle pain, fatigue, rhinitis, sore throat, headache, anorexia, watery diarrhea with no apparent cause, acute confusion, sudden fall with no apparent cause; OR - worsening of chronic respiratory symptoms (COPD, asthma, chronic cough, ...). | **Prioritizing testing** for possible cases (with particular attention to health care staff, occupants/staff in residential communities) and high-risk contacts who are professionally in contact with people at risk of developing a serious form of the disease.  **If capacity permits,** a test can be performed for any person requiring hospitalization or any new resident of a residential entity. |
| 15/05 | **Possible case:** A person with  - at least one of the following main symptoms, with acute onset and without obvious other cause: cough, dyspnea, thoracic pain, acute anosmia or dysgeusia; OR  - at least two of the following symptoms, without obvious other cause: fever, muscle pain, fatigue, rhinitis, sore throat, headache, anorexia, watery diarrhea, acute confusion, sudden fall; OR - worsening of chronic respiratory symptoms (COPD, asthma, chronic cough, ...), without obvious other cause.  **Radiologically confirmed case**: A radiologically confirmed case is a person in whom the RT-PCR test for COVID-19 is negative, but in whom the diagnosis of COVID-19 is made based on a suggestive clinical presentation AND a compatible CT thorax.  **Confirmed case**: A person where the diagnosis of COVID-19 infection is done by a molecular test. | **Rapid Antigen** **testing** and **serology** are used in specific situations from 15/05 and 20/05 respectively. |
| 12/06 |  | *Added to* ***Prioritizing testing***: All high-risk contacts are tested. |
