# Supplementary material for: All-cause mortality supports the COVID-19 mortality in Belgium and comparison with major fatal events of the last century
Source: Arch Public Health. 2020 Nov 13;78:117. doi: 10.1186/s13690-020-00496-x (PMC7662738; doi:10.1186/s13690-020-00496-x)
Supplement: Supplementary file 2 — Additional file 2: Supplementary Table 2. COVID-19 and all-cause mortality for 0-64, 65-84 and 85+ age groups, March 20th to April 28st 2020, Belgium. Supplementary Fig. 1. Mortality all-cause (Be-MOMO) and related to COVID-19 for 0–64, 65–84 and 85+ age groups, Belgium. [file 13690_2020_496_MOESM2_ESM.docx]

All-cause mortality supports the COVID-19 mortality in Belgium and comparison with major fatal events of the last century.

Additional file 2

Supplementary Table 2. COVID-19 and all-cause mortality for 0-64, 65-84 and 85+ age groups, March 20^th^ to April 28^st^ 2020, Belgium

| Age group (years) | Deaths from all causes observed | Deaths from all causes expected | Deaths from all causes in excess | % excess mortality | COVID-19 deaths (% lab-confirmed) | | %  COVID-19 by number of deaths from all causes in excess | |
| --- | --- | --- | --- | --- | --- | --- | --- | --- |
| 0-64 | 2,081 | 1,727 | 354 | 20.5% | 406 | (80%) | 115% |  |
| 65-84 | 8,441 | 5,087 | 3,354 | 65.9% | 3,069 | (72%) | 92% |  |
| 85+ | 9,637 | 5,387 | 4,250 | 78.9% | 4,088 | (61%) | 96% |  |
| Unknown |  |  |  |  | 13 | (15%) |  |  |
| **Total** | **20,159** | **12,242** | **7,917** | **64.7%** | **7,576** | **(66%)** | **96%** |  |


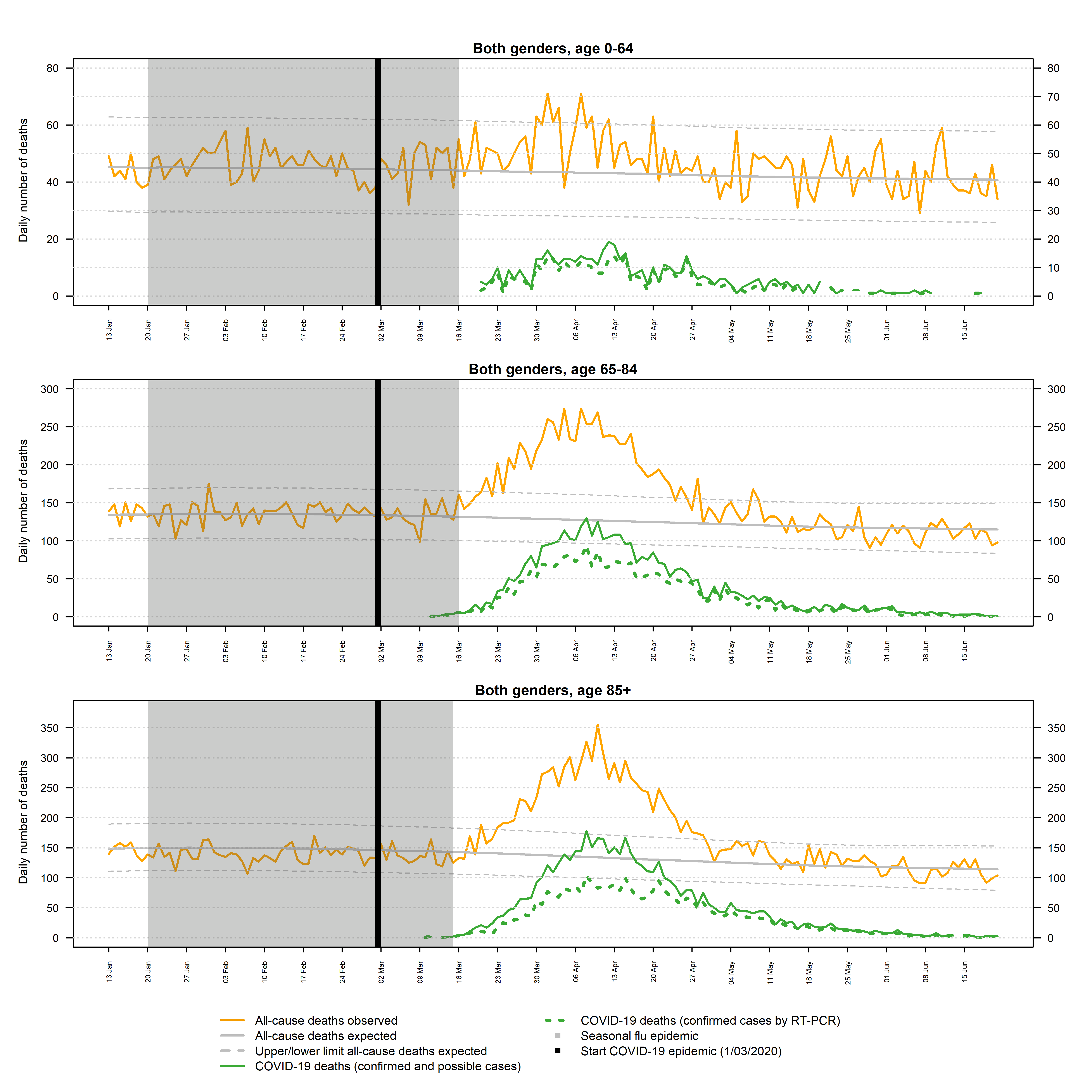


Supplementary Figure 1. Mortality all-cause (Be-MOMO) and related to COVID-19 for 0-64, 65-84 and 85+ age groups, Belgium

*How to read this graph? When the number of deaths per day (orange line) exceeds the upper or lower limits of the deaths predicted by the modelling (grey dashed lines), there is a significant excess or under-mortality. The green curve corresponds to the daily number of COVID-19 deaths (all diagnostic status and all place of death). The green dotted line represents laboratory-confirmed COVID-19 deaths (all places of death).*
